# Therapeutic Targeting of the Galectin-1/miR-22-3p Axis Regulates Cell Cycle and EMT Depending on the Molecular Subtype of Breast Cancer

**DOI:** 10.3390/cells14040310

**Published:** 2025-02-19

**Authors:** Ju Yeon Kim, Jun Ho Lee, Eun Jung Jung, Young Sim Son, Hee Jin Park, Jae Myung Kim, Taejin Park, Sang-Ho Jeong, Jinkwon Lee, Tae Han Kim, Seon Min Lee, Jeong Doo Heo

**Affiliations:** 1 Department of Surgery, Gyeongsang National University Hospital, Gyeongsang National University College of Medicine, Jinju 52727, Republic of Korea; juyeon0910@hanmail.net (J.Y.K.); frontier-jini@hanmail.net (H.J.P.); jmjidia@hanmail.net (J.M.K.); 2 Institute of Health Science, Gyeongsang National University College of Medicine, Jinju 52727, Republic of Korea; ysson0301@gmail.com (Y.S.S.); taejinpark@gnu.ac.kr (T.P.); shjeong@gnu.ac.kr (S.-H.J.); 3 Department of Surgery, Changwon Hanmaeun Hospital, Hanyang University College of Medicine, Changwon 51139, Republic of Korea; junho1231.lee@gmail.com; 4 Department of Surgery, Gyeongsang National University Changwon Hospital, Gyeongsang National University College of Medicine, Changwon 51472, Republic of Korea; bigcap77@gmail.com (J.L.); taehan.email@gmail.com (T.H.K.); 5 Gyeongnam Bio-Health Research Support Center, Gyeongnam Branch Institute, Korea Institute of Toxicology (KIT), Jinju 52834, Republic of Korea; smlee84@kitox.re.kr (S.M.L.); jdher@kitox.re.kr (J.D.H.)

**Keywords:** galectin-1, microRNA-22-3p, breast cancer, molecular type, cell cycle, epithelial–mesenchymal transition

## Abstract

Breast cancer is a highly heterogeneous disease; hence, it is crucial to understand its biology and identify new targets for the development of effective treatments. Galectin-1 is known to play an oncogenic role in breast cancer progression. It is known that oncogenic factors can influence cancer progression through interactions with miRNAs. The purpose of this study is to identify the clinical significance and biological role of galectin-1 and miR-22-3p in cancer progression according to the molecular subtype of breast cancer. We analyzed the expression of galectin-1 and miR-22-3p using cancer tissues and the correlation with clinical pathological characteristics. In addition, we investigated the regulation of the cell cycle and EMT processes of cancer progression through the galectin-1/miR-22-3p axis using cell lines of different breast cancer subtypes. miR-22-3p negatively regulates galectin-1 expression and the two molecules have opposite patterns of oncogenic and tumor-suppressive functions, respectively; furthermore, these two molecules are associated with metastasis-free survival. Cell experiments showed that miR-22-3p overexpression and galectin-1 knockdown inhibited the proliferation and invasion of breast cancer cells. Galectin-1 regulates different cancer progression pathways depending on the molecular subtype. In hormone receptor-positive breast cancer cells, galectin-1 knockdown mainly inhibited cell cycle-related substances and induced G0/G1 arrest, whereas in triple-negative breast cancer cells, it suppressed molecules related to the epithelial–mesenchymal transition pathway. In conclusion, the miR-22-3p/galectin-1 axis regulates different cancer metastasis mechanisms depending on the specific molecular subtype of breast cancer, and miR-22-3p/galectin-1 axis modulation may be a novel target for molecular subtype-specific personalized treatment.

## 1. Introduction

Breast cancer is a common female cancer with a high incidence worldwide and is an important cause of mortality in women. However, the development of screening test programs and the advancement of adjuvant treatment have led to an improved survival rate [1]. Various types of customized breast cancer treatments are currently being developed based on molecular genetic techniques [2]. A better understanding of the biology of highly heterogeneous breast cancer and the identification of new targets are crucial for developing effective treatments [3].

MicroRNAs (miRNA or miR) are small non-coding RNAs (ncRNAs) found in plants, animals, and viruses. They consist of 22 nucleotides and regulate RNA silencing and post-transcriptional gene expression [4,5]. miRNAs have emerged as promising and novel intervention tools for cancer management [6]. These small regulatory ncRNAs are widely deregulated in many types of human cancers, including breast cancer, and are associated with tumorigenesis and tumor progression [7,8]. Currently, many studies are being conducted on miRNAs regarding their role in cancer diagnosis and their therapeutic usefulness [9]; thus, it is essential to find a functional target to identify the role of candidate miRNAs [10,11]. Hundreds of targets can be identified by analyzing the complementarity between the 3′ UTR of the target gene and the seed sequence of the miRNA using the target search program [12]. Out of the hundreds to thousands of search targets, it is a time- and effort-consuming process to find a functional miRNA target that serves as a diagnostic and therapeutic target for cancer. Therefore, proteomic markers identified in cancer tissues as the functional targets of miRNAs may be useful for identifying more specific targets [13].

Breast cancer-specific proteins have been previously identified through the proteomic analysis of breast cancer and normal breast tissues [14,15]. It was found that galectin-1 expression was higher in breast cancer tissues than that in normal tissues, and its overexpression was correlated with advanced stage and lymph node metastasis. Galectins belong to the lectin family and share a common affinity for β-galactoside. The first family member to be described was galectin-1. Human galectin-1 is encoded by the LGALS1 gene located at chromosome 22q13.1. It is a homodimeric protein with a single carbohydrate-recognition domain of 134 amino acids [16,17]. Galectin-1 shows increased expression in various carcinomas, and this is associated with a poor prognosis [18,19,20,21,22,23,24,25,26]. In breast cancer, galectin-1 is involved in the sensitivity to radiation and chemotherapy, tumor progression, and metastasis [26,27,28,29]. Despite the conflicting results reported for several cancers, it has been established that galectin-1 plays an oncogenic role in the tumor progression of breast cancer. Therefore, the discovery of miRNAs that regulate the expression of galectin-1 may also play a role in revealing possible therapeutic targets. miR-22-3p was identified as a candidate that regulates galectin-1 based on the direct binding detected between the two through the target scan program.

MiR-22-3p is expressed in several cancers; its expression increases or decreases depending on the cancer type and plays an essential role in various physiological processes and cancers via multiple targets [30,31,32,33,34,35,36,37,38,39]. Few studies have been conducted on miR-22-3p that regulates galectin-1 in cancer. The aim of this study is to identify the expression of galectin-1 and miR-22-3p in breast cancer and analyze their value as prognostic factors, to elucidate the role of the miR-22-3p/galectin-1 axis in the cell cycle and epithelial–mesenchymal transition (EMT) pathway according to molecular subtypes of breast cancer, and to determine whether the modulation of the galectin-1/miR-22-3p axis could be a molecular subtype-specific breast cancer treatment strategy.

## 2. Materials and Methods

### 2.1. Tissue Samples

Fifty-four patients who underwent curative surgery for breast cancer at Gyeongsang National University Hospital between August 2007 and December 2011 were randomly selected from the institutional database. Ethical approval was obtained from the Institutional Review Board of Gyeongsang National University Hospital (GNUHIRB2013-5219). Patients with intraepithelial ductal carcinoma, squamous cell carcinoma, metastatic carcinoma, and malignant phyllode tumor were excluded from the study. Of the 54 patients with breast cancers, 4 had medullary carcinoma, 2 had invasive lobular carcinoma, and 48 had invasive ductal carcinoma, not otherwise specified. During surgery, the cancerous lesion and normal tissue adjacent to the tumor were resected and stored at −70 °C until further analysis. The cancer lesion was obtained from the center of the cancer by the pathologist and normal control breast tissue adjacent to the tumor was harvested from the edge of the resection after a negative resection margin was confirmed using frozen biopsy in patients who underwent breast-conserving surgery. Tissues from patients with a positive resection margin in the final pathologic report were excluded. In patients who underwent a total mastectomy, two pieces of tissue were obtained from the normal lesion site at least 3 cm away from the cancerous tissue. One piece was stored and the other was stained for the presence of cancer cells; only the normal tissue adjacent to the tumor that was confirmed to be free of cancer cells was used for testing. Pathological and follow-up data were reviewed. Distant metastases were assessed and patients with local breast recurrence, axillary lymph node recurrence, and newly diagnosed contralateral breast cancer were excluded from the study. The time of metastasis was defined as the date of confirmation via biopsy or imaging. Metastasis-free survival (MFS) was defined as the period from the date of breast cancer surgery to the date of the first biopsy- or imaging-confirmed diagnosis with distant metastasis, or the last follow-up.

### 2.2. Cell Culture

The following human breast cancer cell lines obtained from the Korean Cell Line Bank (KCLB; Seoul, Republic of Korea) were used in the study: MCF-7, T47D, ZR-75, HCC 1954, SK-BR-3, MDA-MB 231, and HCC 70. The HEK293A (human embryonic kidney cells) cell line was purchased from Thermo Fisher (Cat. R70507), and the MCF-10A (human normal mammary epithelial cells) cell line was purchased from the American Type Culture Collection (ATCC; CRL-10317). Most cell lines were maintained in RPMI-1640 medium supplemented with 10% fetal bovine serum, 100 U/mL penicillin, and 100 μg/mL streptomycin (all from Gibco, Baltimore, MD, USA) and cultured at 37 °C in a humidified atmosphere of 5% CO_2_. The MCF-10A cells were cultured in DMEM/F12 supplemented with 5% horse serum, 20 ng/mL hEGF, 0.5 mg/mL hydrocortisone, 100 ng/mL cholera toxin, 10 μg/mL insulin, and 1% Pen/Strep.

### 2.3. miR-22-3p Mimics (miR-22-3p), miRNA-22-3p Inhibitor (Anti-miR-22-3p), and Short Interfering RNA (siRNA) Transfection

For transient transfection, hsa-miR-22-3p mimics (miR-22-3p; 5′-AAGCUGCCAGUUGAAGAACUGU-3′, TaqMan Gene Expression Assay ID PM10203) or its control miRNA (miR-CTL; Cat. No. AM17110) and a hsa-miR-22-3p inhibitor (anti-miR-22-3p; AAGCUGCCAGUUGAAGAACUGU, assay ID AM10203) or its control anti-miRNA (anti-miR-CTL; Cat. No. AM17010) were purchased from Ambion (Austin, TX, USA). The control siRNA (siCTL; Cat. No. sc-37007) and galectin-1-targeting siRNAs were obtained from Santa Cruz Biotechnology (Santa Cruz, CA, USA). The galectin-1-targeting siRNA (sigalectin-1; sc-35441) used in the study was a pool of three different siRNAs (sc-35441A 5′-CAGCAACCUGAAUCUCAAATT-3′, sc-35441B 5′-CCAGAUGGAUACGAAUUCATT-3′, and sc-35441C 5′-GUGUGGCCUUUGACUGAAATT-3′). Breast cancer cells were transfected with specific RNA using the Lipofectamine 2000 reagent (Invitrogen, Carlsbad, CA, USA) according to the manufacturer’s instructions. The transfection concentration was applied from 50 to 200 nM. After 6 h (for miR-22-3p-targeting precursors) or 16 h (for galectin-1-targeting siRNAs), the transfection medium was replaced with a fresh complete medium. Transfection efficiency was confirmed by real-time reverse transcription-polymerase chain reaction (qRT-PCR).

### 2.4. Establishing a Stable Knockdown Cell for Galectin-1 Using Short-Hairpin RNA (shRNA) and Stable Overexpressing Cell for miR-22-3p

To establish a stable galectin-1 knockdown cell, breast cancer cells were transfected with a galectin-1-targeting shRNA plasmid (shgalectin-1, Sigma-Aldrich, St. Louis, MO, USA) and the non-target shRNA control plasmid (sheGFP, Sigma-Aldrich). The following two shRNA sequences were designed for the knockdown of human galectin-1 expression: 5′-CGGCAACCTGTGCCTGCACTTCAACTCGAGTTGAAGTGCAGGCACAGGTTGTTTTTTG-3′ and 5′-CCGGCGCTAAGAGCTTCGTGCTGAACTCGAGTTCAGCACGAAGCTCTTAGCGTTTTTG-3′. The sequence used for *sheGFP* was 5′-CGGCGTCTTTCTATCCATCGAATTCTCGAGAATTCGATGGATAGAAAGACGTTTTTG-3′. A total of 1 μg of galectin-1-targeting shRNA or shRNA control plasmid DNA were transfected into T47D and MDA-MB-231 cells using Lipofectamine 2000 (Invitrogen) according to the manufacturer’s instructions. Stable shRNA-expressing cell lines were selected by puromycin (1 μg/mL). Galectin-1 expression was confirmed by Western blot. For stable overexpression of miR-22-3p, hsa-miR-22-3p (miRBase accession ID: MI0000078) was amplified using primers FR: GGGGGATCCCTGGGGCAGGACCCT and RP: GGGGAATTCAACGTATCATCCACCC, and was cloned into the BamHI and EcoRI sites of the pCMV-tag2B vector (Stratagene, LA Jolla, CA, USA). The sequences of all the clones were confirmed by sequencing. Cell lines were transfected with 1 μg of the miR-22-3p-expressing vector (pCMV-tag2B/miR-22-3p) or a negative control vector (empty vector, pCMV-tag2B) using Lipofectamine 2000 (Invitrogen). In order to produce a stable miRNA overexpression pool, the cells were selected using puromycin (Sigma-Aldrich, 1–2 μg/mL) for 1–2 days, and then the single cell colonies were transferred onto a 96-well plate. The cells were maintained by adding 50% fresh medium every 3–4 days for 2–3 weeks. miR-22-3p expression was confirmed by qRT-PCR.

### 2.5. Luciferase Reporter Assay

Luciferase reporter assays were performed using the Luciferase Reporter Assay System (Promega, Fitchburg, WI, USA) according to the manufacturer’s instructions. Probable target binding sites of miR-22-3p within 3′ UTR of galectin-1 were determined from the Targetscan databases: “www.Targetscan.org (accessed on 11 February 2021)”. The 3′ UTR binding lesion of galectin-1 was amplified from human genomic DNA and cloned into the XbaI/BamHI sites of the pGL3 control vector (Promega). The galectin-1 3′ UTR-mutant construct was generated using a QuikChange XL Mutagenesis Kit (Stratagene). Wild-type and mutant sequences were confirmed by sequencing. For luciferase assays, the cells were seeded into 24-well plates at 5 × 10^4^ cells per well, incubated for 24 h, and then co-transfected with 400 ng of galectin-1 3′ UTR wild-type or mutant plasmids, 80 ng of pRL-TK Renilla luciferase reporter, and 50 nM of control miRNA or pre-miR-22-3p. After 48 h, luciferase activity was measured using the Dual-Luciferase Reporter Assay System (Promega). The luminescent signals were quantified using a luminometer (Glomax; Promega) and those from firefly luciferase were normalized to those from Renilla luciferase. All the assays were performed in triplicate and repeated at least thrice.

### 2.6. RNA Immunoprecipitation Assay

The RNA immunoprecipitation (RIP) assay was carried out employing the Magna RIP RNA-Binding Protein Immunoprecipitation Kit (Millipore, Billerica, MA, USA). The cells were seeded at 5–7 × 10^6^ cells and then transfected with either miR-22-3p mimics or its control miRNA for 48 h at 37 °C. After the transfection period, the cells were lysed using RIP lysis buffer and incubated with magnetic beads conjugated with antibodies against IgG (Abcam, Cambridge, UK; ab172730, 1:50) and AGO2 (Abcam, ab186733, 1:50). The magnetic beads were washed several times with RIP wash buffer and incubated for 30 min at 55 °C with 0.5 mg/mL proteinase K buffer to degrade the proteins associated with RNA. The precipitated RNA was then extracted from the beads using TRIzol reagent (Invitrogen) and analyzed using qRT-PCR.

### 2.7. Cell Proliferation Assay

The human breast cancer cells were seeded into 24-well plates at 2 × 10^4^ cells/well in a complete medium and incubated for 24 h. After transfection with miRNAs or siRNA, the cells were changed to a fresh medium containing 10% FBS and 1% Pen/Strep. The cells were incubated for 24, 48, 72, or 96 h, as indicated, and then subjected to the MTT assay. In brief, the cells were incubated with 0.5 mg/mL of 3-(4,5-dimethyl-thia-zol-2-yl)-2,5-diphenyltetrazoliumbromide (MTT, Sigma) solution for 3 h. The supernatants were carefully removed, and dimethyl sulfoxide (DMSO) was added to dissolve the purple formazan crystals. Absorbance was measured at 570 nm using a VersaMax ELISA Microplate Reader (Molecular Devices, San Jose, CA, USA). All the experiments were repeated thrice.

### 2.8. Colony Forming Assay

The cells were seeded into 6-well tissue culture dishes at 5 × 10^2^ cells/well in triplicate, and the plates were incubated at 37 °C for 10–15 days. The medium was changed every 3 days. The number of colonies per dish was counted after staining with crystal violet (1%, *w*/*v*; Sigma) for 1–3 h at room temperature.

### 2.9. Wound Healing Assay

The wound-healing experiment was performed using a 2-well silicone insert (Ibidi, Martinsried, Germany). Culture inserts were transferred to 12-well plates with sterile tweezers. The MDA-MB 231 and T47D cells were seeded into 6-well plates at 5–7 × 10^5^ cells/well, incubated for 24 h, and transfected with constructs encoding miRNAs or siRNAs. The cells were cultured for 48 h in a humidified incubator at 37 °C and 5% CO_2_. The cells were then reseeded into 2-well silicone inserts with 70 μL cell suspension (approximately 5 × 10^5^ cells/insert side) and incubated for 24 h. Mitomycin C (10 μg/mL; Sigma-Aldrich) was added 2 h before the inserts were removed. The inserts were gently removed and examined under a Nikon ECLIPSE Ti inverted microscope (Nikon Instruments Inc., Tokyo, Japan) at time zero. The plates were washed with PBS and incubated with a new complete medium containing 10% serum for 24–120 h. At different time points, the plates were observed under a microscope (40× magnification), and the wound site was measured to reflect cell migration. The relative percentages of wound closure were determined using ImageJ software version 1.52d (NIH). All the data were normalized to the mean value at time zero.

### 2.10. Invasion Assay

For the invasion assay, transwell inserts (8-μm-pore size, 6.5 mm diameter; Costar, Cambridge, MA, USA) were coated with BD Matrigel Basement Membrane Matrix (BD Biosciences, Bedford, MA, USA) for 2–4 h in a humidified 37 °C incubator under 5% CO_2_. Next, the transfected cells (3–7 × 10^4^ per well) were seeded with 250 μL of the serum-free medium in the upper chamber, and 600–750 μL of the complete medium with 10% FBS was loaded into the basal chamber. The plates were incubated at 37 °C for 22–72 h, and the non-invaded cells were scraped off with a cotton swab. The translocated cells (those on the bottom of the upper chamber membrane) were fixed with 4% formaldehyde for 20 min at room temperature and the cells were stained with DAPI at 37 °C for 10–30 min. Using a microscope (Nikon ECLIPSE Ti inverted microscope), the migrated cells were counted in five randomly selected microscopic fields at 100× magnification.

### 2.11. Western Blotting

Proteins from the transfected cells were extracted using RIPA lysis buffer containing protease inhibitors (Sigma, St. Louis, MO, USA). The lysate concentrations were estimated by the Bradford assay (Bio-Rad, Hercules, CA, USA), and 30 µg of the total protein mixed with 5 × SDS Laemmli buffer was loaded in each lane. The proteins in the lysates were separated using 10–15% SDS-PAGE and transferred to PVDF membranes (Invitrogen). The membranes were incubated with 5% skim milk in TBST for 1 h at room temperature and incubated with primary antibodies (Supplementary Table S1) overnight at 4 °C. A peroxidase-conjugated secondary antibody was used to detect immunoreaction.

The immunocomplexes were detected using an ECL detection kit (Thermo Fisher Scientific, Waltham, MA, USA). The protein expression levels were analyzed using the image analysis software Image-J (NIH).

### 2.12. RNA Extraction and qRT-PCR

Total RNA was isolated from the cell lines and breast tissues using the QIAzol reagent (Qiagen, Valencia, CA, USA) according to the manufacturer’s instructions. For the qRT-PCR of mature miRNAs, the cDNA synthesis of specific miRNAs was performed using the TaqMan MicroRNA Reverse Transcription Kit (Thermo Fisher Scientific, Waltham, MA, USA) with miRNA-specific primers (TaqMan microRNA Assay; has-miR-22-3p, 000398; U6 snRNA, 001973). Subsequently, quantitative real-time PCR was performed using the TaqMan MicroRNA Assay Kit (Applied Biosystems) in accordance with the manufacturer’s instructions. For the mRNA expression analysis of specific genes, cDNA synthesis was performed using the Superscript III cDNA synthesis kit (Invitrogen). qRT-PCR was performed in a PCR reaction mixture using specific primers for the 20 × TaqMan Gene Expression Assay and 2 × TaqMan Gene Expression Master Mix (#4369016; Applied Biosystems). Specific primers for TaqMan Gene Expression Assay were purchased from Applied Biosystems. The following primers were used: Galectin-1 (assay ID: Hs00355202_m1), Vimentin (assay ID: Hs00185584_m1), Snail (assay ID: Hs00195591_m1), Slug (assay ID: Hs161904_m1), and GAPDH (assay ID: Hs02758991_g). U6 and GAPDH were used as internal controls to normalize the expression of microRNA and mRNA, respectively. The ViiA 7 Real-Time PCR System (Applied Biosystems; Thermo Fisher Scientific, Inc.) was used to measure the relative expression levels. The differential expression level was calculated using the 2^−ΔΔCt^ formula.

### 2.13. Animal Experiments

Athymic (nude, 5-week) female mice were obtained from KOATECH (Pyeongtaek, Republic of Korea) and acclimatized for at least 1 week before the beginning of the study. The animal experiments were conducted with the permission and supervision of the Animal Ethics Committee of Gyeongnam Bio-Health Research Support Center, Korea Institute of Toxicology (KIT) (IACUC approval number: 15-1-0003). Xenografts were established from the empty vector (N = 5), miR-22-3p-overexpressing clones (N = 5), and galectin-1 knockdown clones (N = 5) for MDA-MB 231 cells by subcutaneous implantation of 5 × 10^6^ cells in serum-free media into the flank of each mouse. After 4–5 weeks, the mice were euthanized. Tumor volume was calculated using the following formula: [V = Length × (Width/2)^2^ × 3.14].

### 2.14. Flow Cytometry

After transient transfection with sigalectin-1, miR-22-3p, and each control group for 48 h, the transfected cells were prepared. The cells were collected after exposure to a 0.25% trypsin-EDTA solution for 5 min. After centrifugation at 1500 rpm for 5 min, the cells were washed twice in cold PBS and fixed with cold ethanol (70% by weight) at −20 °C overnight. For analysis, the cells were stained with PBS containing 0.1% Triton X-100, RNase A (10 g/mL; Fermentas, Burlington, ON, Canada), and propidium iodide (PI) (50 g/mL; Sigma-Aldrich, St. Louis, MO, USA). All the samples were incubated at room temperature for 30 min in the dark. Flow cytometry (LSRFortessa™ X-20, BD Biosciences) measurements were performed according to the standard procedure, and the data obtained using the BD FACSDiva software (version 8.0.3, BD Biosciences) were analyzed.

### 2.15. Statistical Analysis

Statistical analyses were performed using SPSS (version 19.0; IBM Corp., Armonk, NY, USA). The data are presented as mean ± SD. Student’s *t*-test was used to compare the differences between the two groups, while one-way ANOVA (including Dunnett’s test) was used to compare the differences among multiple groups. The prognostic significance of the factors was determined using the Kaplan–Meier method with a log-rank test. Statistical significance was set at *p* < 0.05.

## 3. Results

### 3.1. Clinical Relevance of Galectin-1 Expression in Breast Cancer

**Figure 1:**
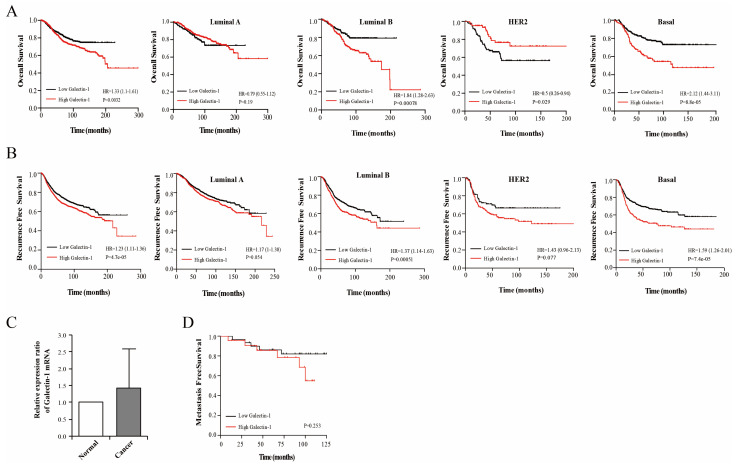
Survival analyses regarding galectin-1 expression in the breast cancer cells. Analyses of the (**A**) overall survival (OS) and (**B**) recurrence-free survival (RFS) rates according to the galectin-1 expression levels using the online KM-Plotter database. The survival curve according to the breast cancer molecular type (luminal A and B, Her-2 enriched, and basal type) was analyzed in the high- and low-expression groups of galectin-1. (**C**) Measurement of the galectin-1 mRNA expression levels using qRT-PCR in the breast cancer and noncancerous breast tissues. The expression ratio of galectin-1 was higher in the cancer tissues than in the normal tissues (normal vs. cancer tissues; 1 vs. 1.42 ± 1.18, *p* = 0.01). (**D**) The high galectin-1-expression group shows a lower metastasis-free survival rate than the low galactin-1 expression group; however, this difference was not significant. qRT-PCR, real-time reverse transcription-polymerase chain reaction.

Survival analyses based on galectin-1 expression were performed using the online KM-Plotter database: “https://kmplot.com (accessed on 14 March 2023)”. The overall survival (OS) and recurrence-free survival (RFS) rates were calculated based on the gene expression data and survival information. The high galectin-1 expression group showed lower OS and RFS than the low-expression group (*p* = 0.0032, 4.7 × 10^−0.5^). Subgroup analyses showed significantly lower survival rates in the patients with high galectin-1 expression in the luminal B and basal types of breast cancer (Figure 1A,B). The significance of the survival rate was higher in the basal type group than in the other subtypes. Galectin-1 expression was measured using RT-PCR in 54 breast cancer tissues and normal control breast tissues. However, because of an RNA quantity problem in one sample, the analysis was conducted on the remaining 53 cancer tissue samples. The mean level of galectin-1 was higher in the cancer tissues than in the normal tissues (Figure 1C, normal vs. cancer tissues; 1 vs. 1.42 ± 1.18, *p* = 0.01). The high galectin-1 expression group had a lower MFS rate than the low galectin-1 expression group; however, this difference was not significant (Figure 1D, *p* = 0.253).

### 3.2. Galectin-1 Knockdown Inhibits Breast Cancer Proliferation and Invasiveness

**Figure 2:**
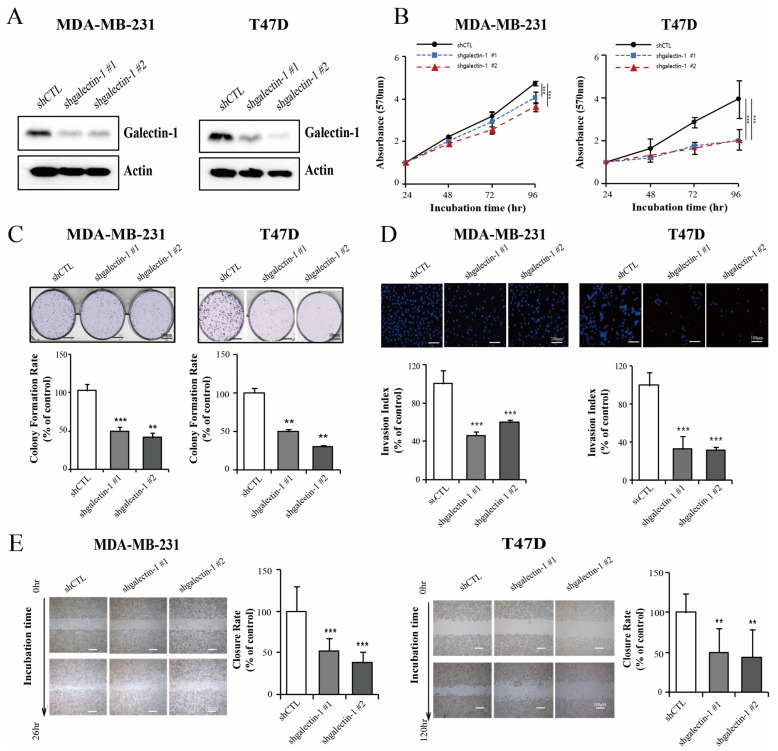
Galectin-1 knockdown inhibits cell proliferation and invasion. (**A**) Determination of galectin-1 expression after transfection with shRNA targeting galectin-1 in the MDA-MB 231 and T47D cells using Western blot. Results of (**B**) cell proliferation, (**C**) colony forming assay, (**D**) invasion assay, and (**E**) wound healing assay in the cancer cells transfected with galectin-1 shRNA or control. Galectin-1 knockdown inhibited cell proliferation, colony formation, invasion, and wound-healing ability. Significance was determined using an unpaired, two-tailed Student’s *t*-test when compared with the control. Data are presented as means ± SD from three independent experiments. ** *p* < 0.01, and *** *p* < 0.001. SD, standard deviation.

The galectin-1 expression levels were significantly associated with OS and RFS in breast cancer. Next, we investigated the effects of galactin-1 on cell proliferation, colony formation, invasion, and wound healing in triple-negative and hormone-positive cells, such as the MDA-MB-231 and T47D cells. We confirmed the galectin-1 silencing ability by the Western blot analysis of the control (denoted as shControl) and galectin-1 knockdown cells (denoted as shgalectin-1) (Figure 2A) and selected two shgalectin-1 from each cell line for further functional analysis. Stable knockdown of galectin-1 induced a decrease in cell proliferation compared with that in shControl in the MDA-MB 231 cell (*p* < 0.001; Figure 2B). In addition, the suppression of galectin-1 induced a decrease in cell proliferation compared with that in the control group in the T47D cells (*p* < 0.001; Figure 2B). These results were similar to the colony-formation abilities of the MDA-MB 231 and T47D cells as the galectin-1 knockdown cells showed a significant decrease in colony formation in comparison with that in the control (*p* < 0.01 and *p* < 0.01, respectively; Figure 2C). The number of invaded cells was significantly lower in the galectin-1 knockdown group than in the control group in both the MDA 231 (*p* < 0.001; Figure 2D) and T47D cells (*p* < 0.001; Figure 2D). In the wound healing assay, the galectin-1-knockdown MDA-MB 231 cells exhibited a slower healing rate than the control cells at 26 h (*p* < 0.001; Figure 2E) and at 120 h in the T47D cells (*p* < 0.01; Figure 2E).

### 3.3. Galectin-1 Is the Direct Target Gene for miR-22-3p

**Figure 3:**
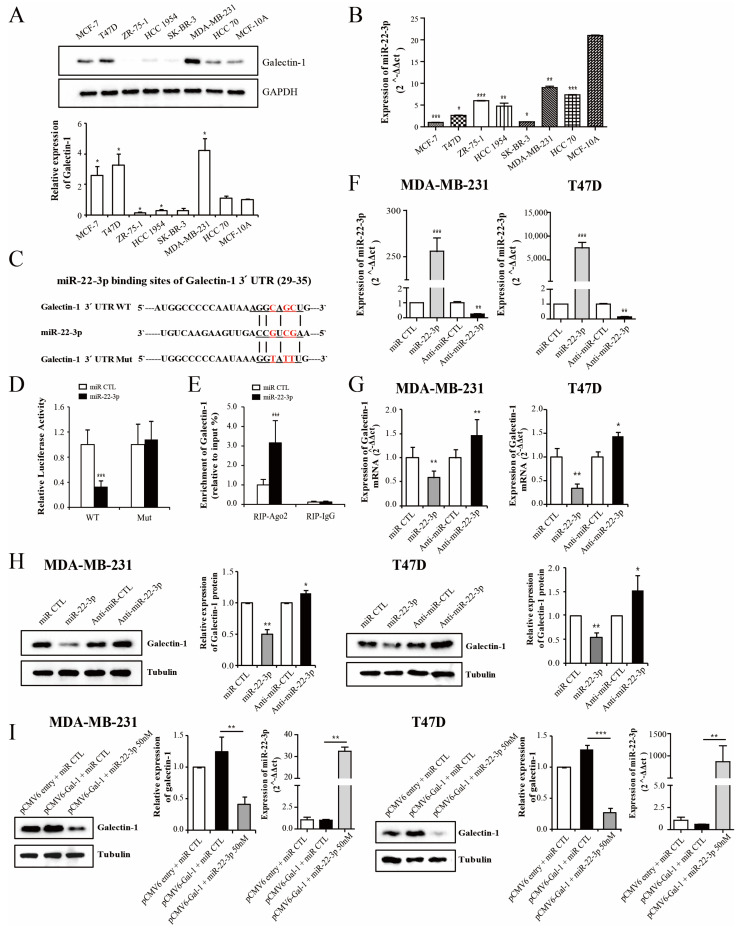
Galectin-1 is the direct target gene for miR-22-3p. (**A**,**B**) Analysis of the expression patterns of galectin-1 and miR-22-3p according to breast cancer cell types using qRT-PCR and Western blot. The expression of galectin-1 and miR-22-3p varies depending on the molecular subtype of breast cancer. (**C**) Representative diagram indicating the conserved binding site of miR-22-3p in the 3′ UTR of galectin-1 mRNA. (**D**) Relative luciferase activity of galectin-1 measured 48 h after the co-transfection of wild or mutant galectin-1 3′ UTR reporter genes with miR-22-3p in the HEK293A cells. The luciferase activity was normalized to *Renilla* luciferase activity. (**E**) RNA immunoprecipitation assay was performed to further determine the galectin-1 expression levels in the HEK 293A cells transfected with miR-22-3p mimic or its control miRNA. (**F**,**G**) Levels of miR-22-3p and galectin-1mRNA determined using qRT-PCR after transfection with miR-22-3p mimics (miR-22-3p) or its control miRNA (miR-CTL), and miR-22-3p inhibitor (anti-miR-22-3p) or its control anti-miRNA (anti-miR-CTL) in the MDA-MB 231 and T47D cells. (**H**) Galectin-1 protein level measured using Western blot. (**I**) Galectin-1 level after the transfection of miR-22-3p into the galectin-1 overexpression cells (pCMV6-Gal-1). The galectin-1 expression level was significantly reduced by the transfection of miR-22-3p in the galectin-1 overexpression cells of the MDA-MB-231 and T47D cell lines. Significance was determined using an unpaired, two-tailed Student’s *t*-test when compared with the control. Data are presented as mean ± SD. * *p* < 0.05, ** *p* < 0.01, and *** *p* < 0.001. qRT-PCR, real-time reverse transcription-polymerase chain reaction; SD, standard deviation.

miRNAs usually function by negatively modulating the expression of their target genes. We used widely accepted prediction algorithms to search for miRNAs to modulate galectin-1, such as TargetScan, PicTar, miRanda, Micro-T, and miRecords. We found that galectin-1 might interact with miR-22-3p, miR-1275, miR-885-3P, and miR-1827. miR-22-3p was commonly found in most programs, and we selected miR-22-3p as the galectin-1 regulatory miRNA for further analyses. The binding site between galectin-1 and miR-22-3p was confirmed using a target scan program. The expression levels of galectin-1 and miR-22-3p varied across different breast cancer cell lines (Figure 3A,B). Galectin-1 expression was higher in the T47D and MDA-MB 231 cells than in the other breast cancer cell lines and in the non-tumor epithelial cell line. miR-22-3p expression was lower in most breast cancer cells than in the MCF-10A cells. According to previous clinical results, increased galectin-1 expression is associated with poor prognosis in patients with either luminal or triple-negative cancer types. We selected two cell lines of the T47D and MDA-MB 231 cells, the luminal type and the triple negative cancer type, respectively, for further functional study. To test whether galectin-1 is a direct target of miR-22-3p, we performed dual-luciferase reporter assays using a fragment of the 3′ UTR of human galectin-1 containing a putative miR-22-3p binding site (galectin-1 3′ UTR WT; Figure 3C). We also created an expression vector encoding a 3′ UTR fragment harboring disruptive mutations at the putative miR-22-3p binding site (galectin-1 3′-UTR MUT; Figure 3C). The galectin-1 3′ UTR WT reporter vectors were co-transfected with miR-22-3p and control miRNA into the HEK 293 A cells. The relative luciferase activity was significantly reduced (*p* < 0.001, Figure 3D). The luciferase activity of the mutated vectors (galectin-1 3′ UTR MUT) was not decreased by the transfection of miR-22-3p (Figure 3D). The RIP assay was performed to further identify the interaction of galectin-1 with miR-22-3p. The results showed that the transfection of miR-22-3p mimics resulted in the enhanced expression of galectin-1 in the Ago2-immunoprecipitation group, suggesting an interaction between miR-22-3p and galectin-1 (*p* < 0.001, Figure 3E). The MDA-MB 231 and T47D cells were transfected with miR-22-3p mimics or its control miRNA, and inhibitors of miR-22-3p (anti-miR-22-3p) or its control anti-miRNA. The qRT-PCR analyses demonstrated that the overexpression of miR-22-3p significantly reduced the mRNA expression of galectin-1 (*p* < 0.01; Figure 3F,G) and that this effect was reversed by the expression of anti-miR-22-3p in both the MDA-MB 231 and T47D cells (*p* < 0.01 and *p* < 0.05, respectively; Figure 3F,G). The protein expression of galectin-1 was measured using Western blot. The overexpression of miR-22-3p significantly reduced the protein levels of galectin-1 (*p* < 0.01; Figure 3H), and the suppression of miR-22-3p by anti-miR-22-3p significantly increased the galectin-1 protein levels (*p* < 0.05; Figure 3H) in both cell lines. To confirm the miR-22-3p effect in the galectin-1 overexpression cells, miR-22-3p was transfected into the galectin-1 overexpression cells of the MDA-MB 231 and T47D cell lines. The overexpression of galectin-1 was significantly reduced by transfection with miR-22-3p in both the MDA-MB 231 and T47D cells (*p* < 0.01 and *p* < 0.001, respectively; Figure 3I). These results suggest that galectin-1 is a direct target of miR-22-3p and that its expression is negatively regulated by miR-22-3p in breast cancer cells.

### 3.4. miR-22-3p Expression Is Associated with the Prognosis in Patients with Breast Cancer

**Figure 4:**
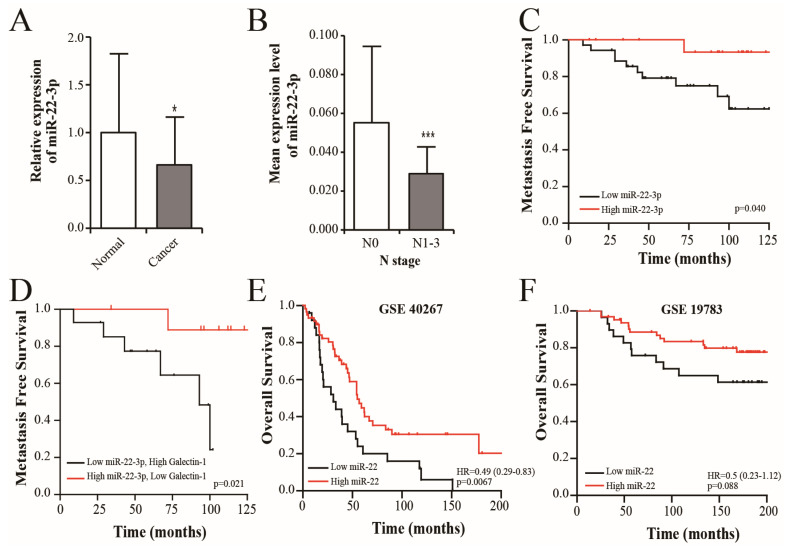
Clinical relevance of miR-22-3p expression in breast cancer. (**A**) Analyses of the miR-22-3p expression levels in 54 pairs of breast cancer and adjacent normal tissues using qRT-PCR. miR-22-3p expression was lower in the cancer tissues compared with that in the normal tissues (1 vs. 0.663, *p* = 0.013). (**B**) The mean miR-22-3p expression levels were significantly lower in the lymph node-positive group (N0 vs. N1-3, *p* = 0.001). (**C**) The Kaplan–Meier curve represents the metastasis-free survival (MFS) rate in patients with breast cancer based on the miR-22-3p expression groups (low or high). The MFS rate for the high miR-22-3p group was higher than that in the low miR-22-3p group (*p* = 0.04). (**D**) The MFS rate was analyzed according to the combined expression levels of galectin-1 and miR-22-3p. MFS for the high miR-22-3p/low galectin-1 group was higher compared with that in the low miR-22-3p/high galectin-1 group (*p* = 0.021). (**E**,**F**) Overall survival analysis using an online database: “https://kmplot.com (accessed on 23 March 2023)”. The high miR-22 expression group had a longer survival time than the low miR-22 expression group (GSE 40267, GSE 19783, *p* = 0.0067 and 0.088, respectively). * *p* < 0.05, and *** *p* < 0.001. qRT-PCR, real-time reverse transcription-polymerase chain reaction.

We measured the expression levels of miR-22-3p in breast cancer tissues using qRT-PCR. In contrast to the galectin-1 expression level, the mean expression level of miR-22-3p was lower in the cancer tissues of the 54 patients with breast cancer than in their normal tissues (*p* = 0.013; Figure 4A). The levels of miR-22-3p expression were not significantly associated with the T stage, hormonal status, or HER-2/neu status. However, the levels of miR-22-3p were lower in the patients with lymph node metastasis than in the patients with negative node metastasis (N0 vs. N1-3 (mean ± SD); 0.0504 ± 0.0386 vs. 0.0284 ± 0.0147; *p* = 0.001), and miR-22-3p expression was negatively correlated with the lymph node metastasis (Table 1, Figure 4B). During the median 135-month follow-up period, 11 patients developed distant metastases. We used the mean value of miR-22-3p to classify patients as “low miR-22-3p” or “high miR-22-3p”. N stage, Her-2/neu status, and miR-22-3p levels were identified as significant prognostic factors. Particularly, patients with low miR-22-3p levels had a lower MFS rate (mean, 105.37 months; 95% confidence interval [CI]: 89.98–120.78) than those with high miR-22-3p levels (mean, 123.3 months; 95% CI: 116.39–130.28) (*p* = 0.04, Table 2 and Figure 4C). Based on the finding that galectin-1 was a direct target of miR-22-3p, the MFS rate was analyzed according to the combined expression levels of galectin-1 and miR-22-3p. The combined expression levels of galectin-1 and miR-22-3p were classified based on their mean expression levels (high miR-22-3p/low galectin-1 vs. low miR-22-3p/high galectin-1). Patients with high miR-22-3p/low galectin-1 expression had significantly longer MFS (mean, 120.89 months; 95% CI: 109.60–132.18) compared with that in the low miR-22-3p/high galectin-1 groups (mean, 78.69 months; 95% CI: 61.61–95.78) (*p* = 0.021, Figure 4D). We further evaluated the relationship between miR-22 expression and the overall survival rate of patients with breast cancer using an online database “https://kmplot.com (accessed on 23 March 2023)”. The survival rates in the two groups were analyzed based on the mean expression of miR-22 (high vs. low expression). The high miR-22 expression group had a significantly longer survival time compared with that of the low miR-22 expression group (*p* = 0.0067 and 0.088, respectively; Figure 4E,F). Similarly to our results, a good prognosis was observed in the high miR-22 expression group in previous studies.

### 3.5. miR-22-3p Inhibits Breast Cancer Cell Proliferation and Invasiveness

**Figure 5:**
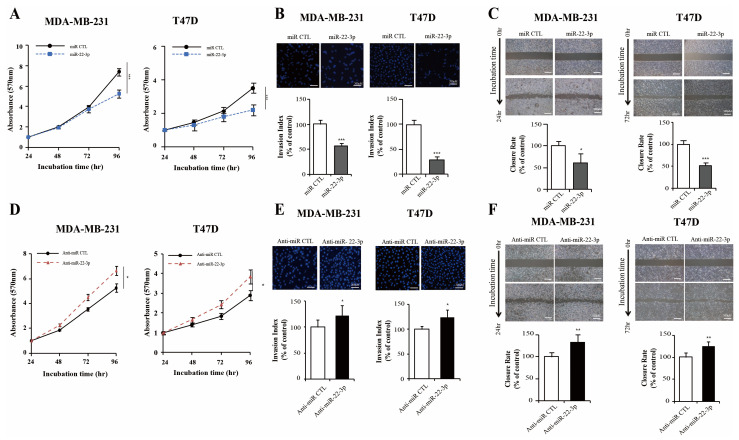
miR-22-3p inhibits cell proliferation, migration, and invasion. The MDA-MB 231 and T47D cells were transfected with miR-22-3p mimics (miR-22-3p) or its control miRNA (miR CTL), and a miR-22-3p inhibitor (anti-miR-22-3p) or its control anti-miRNA (anti-miR-CTL). (**A**,**D**) Cell proliferation assays performed at 24, 48, 72, and 96 h after transfection with miR-22-3p mimics and miR-22-3p inhibitor. miR-22-3p overexpression inhibits cell proliferation, and cell growth was significantly increased after anti-miR-22-3p transfection. (**B**,**E**) Evaluation of invasion activity using a Transwell assay. Fluorescent images of crystal violet immunostaining were obtained and a quantitative assessment was performed. miR-22-3p inhibits the invasive ability of the MDA-MB 231 and T47D cells. Invasion ability was reversed after anti-miR-22-3p transfection. (**C**,**F**) Evaluation of the wound-healing rates at 24 and 72 h after scratching the cell surface with the transfection of miR-22-3p mimics and miR-22-3p inhibitor. The rate of closure between the wound edges is shown. Significance was determined using an unpaired, two-tailed Student’s *t*-test when compared with that in the control. Data are presented as mean ± SD. * *p* < 0.05, ** *p* < 0.01, and *** *p* < 0.001. SD, standard deviation.

As the expression of miR-22-3p in breast cancer tissues is reduced and the survival rate of the group with decreased expression is significantly low, miR-22-3p can be considered a tumor suppressor in breast cancer. We evaluated the biological roles of miR-22-3p in breast cancer using cells transfected with miR-22-3p mimics or its control miRNA and miR-22-3p inhibitor (anti-miR-22-3p) or its control anti-miRNA. We confirmed that cell growth after miR-22-3p transfection was inhibited in both the MDA-MB-231 and T47D cells (*p* < 0.001 and *p* < 0.001, respectively; Figure 5A). Similarly, the invasion abilities of the MDA-MB-231 and T47D cells were significantly inhibited in the miR-22-3p overexpression group compared with those in the control groups (*p* < 0.001 and *p* < 0.001, respectively; Figure 5B). The invasion abilities of the MDA-MB-231 and T47D and cells were also significantly inhibited in the miR-22-3p overexpression group (*p* < 0.05 and *p* < 0.001, respectively; Figure 5C). The effects of miR-22-3p on proliferation, invasion, and wound-healing ability were increased or recovered by anti-miR-22-3p transfection (*p* < 0.05, *p* < 0.05, and *p* < 0.01, respectively; Figure 5D–F). These results demonstrated that miR-22-3p may be a tumor suppressor in breast cancer progression.

### 3.6. Dual Inhibition of Galectin-1 by siRNA and miR-22-3p Showed an Enhanced Antitumor Effect

**Figure 6:**
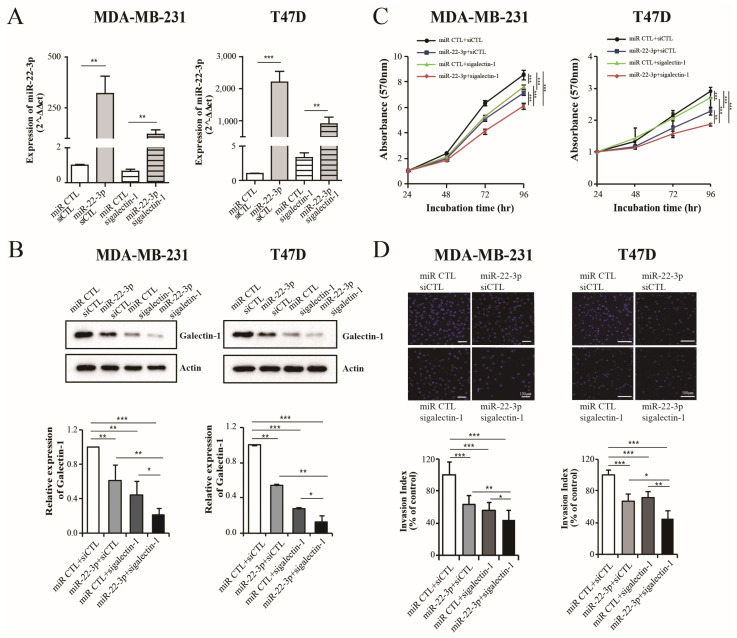
Enhanced antitumor effect on combined treatment with miR-22-3p and sigalectin-1 in breast cancer cells. (**A**,**B**) Analysis of the miR-22-3p and galectin-1 expression levels using qRT-PCR and Western blot after transfection with miR-22-3p, sigalectin-1, or combinations in both the MDA-MB 231 and T47D cells. The combined transfection showed a remarkable reduction in galectin-1 expression. (**C**,**D**) Proliferation and invasion assay after transfection with miR-22-3p, sigalectin-1, or their combination in both the MDA-MB 231 and T47D cells. The combination concentration was set at a 1:1 ratio, 50 nM for Western blot and 100 nM for the proliferation and invasive experiments. Significance was determined using an unpaired, two-tailed Student’s *t*-test when compared with that in the control. Data are presented as mean ± SD. * *p* < 0.05, ** *p* < 0.01, and *** *p* < 0.001. qRT-PCR, real-time reverse transcription-polymerase chain reaction; SD, standard deviation.

We evaluated the antitumor effects of galactin-1 dual inhibition mediated by galectin-1 siRNA and miR-22-3p overexpression. miR-22-3p and galectin-1 siRNAs were co-transfected into the T47D and MDA-MB 231 cells at the concentrations of 50, 100, and 200 nM, which are widely used for miRNA and siRNA transfection. Proliferation and invasion significantly decreased in a dose-dependent manner in both cell lines (Supplementary Figure S1). Therefore, it was predicted that combined transfection would have a synergistic effect, and the combined transfection was performed. The concentration ratio of miR-22 and sigalectin-1 was 1:1, 50 nm for Western blot, and 100 nm for the proliferation and invasion experiments. Indeed, the combined transfection of miR-22-3p and galectin-1 siRNA significantly reduced the galectin-1 expression compared with that after the single transfection of miR-22-3p and galectin-1 siRNA in the MDA-MB-231 and T47D cells (*p* < 0.05 and *p* < 0.05, respectively; Figure 6A,B). These combinations showed the highest inhibitory effect compared with that after single transfection on the proliferation of the MDA-MB-231 (*p* < 0.001; Figure 6C) and T47D cells (*p* < 0.01; Figure 6C). Combined transfection also significantly reduced the invasive capability compared with that after single transfection in the MDA-MB-231 (*p* < 0.05, Figure 6D) and T47D cells (*p* < 0.05, Figure 6D). These results indicated that co-treatment with miR-22-3p and galectin-1 siRNAs enhanced the antitumor effect by increasing galectin-1 suppression in breast cancer cells.

### 3.7. miR-22-3p Overexpression and Galectin-1 Knockdown Regulate Epithelial-to-Mesenchymal Transition (EMT)

**Figure 7:**
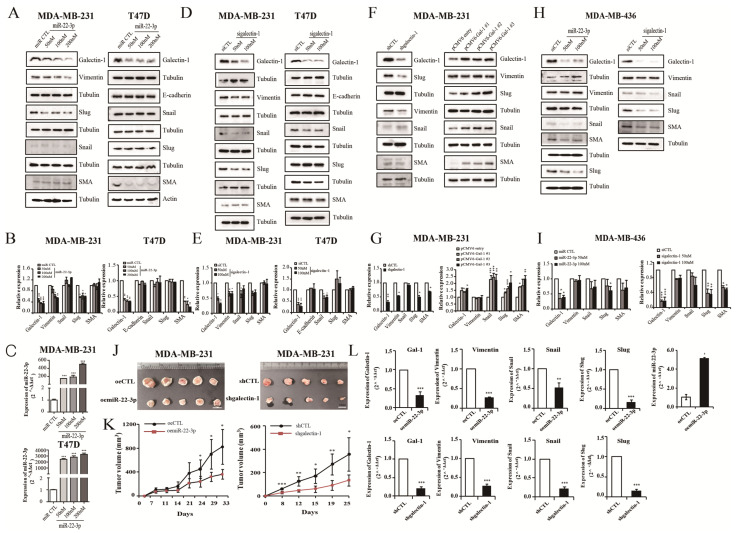
miR-22-3p overexpression and galectin-1 knockdown inhibit EMT progression. Analyses of the expression of EMT-related proteins, Vimentin, Snail, Slug, SMA, and E-cadherin, via Western blot after transfection with miR-22-3p (miR-22-3p overexpression group) and sigalectin-1 (galectin-1 knockdown group). (**A**,**B**) EMT-related proteins were measured in the miR-22-3p overexpression groups of the MDA-MB 231 and T47D cells. (**C**) Transfection efficiency confirmed using miR-22-3p mimic (50, 100, 200 mM) or its control miRNA in MDA-MB 231 and T47D cells. (**D**,**E**) Changes in EMT-related protein in galectin-1 knockdown groups in the MDA-MB 231 and T47D cells. (**F**,**G**) Differential expression of EMT-related proteins in the galectin-1 knockdown group by shgalectin-1 and the galectin-1 overexpression group of the MDA-MB 231 cells. (**H**,**I**) Expression levels of EMT-related proteins in the galectin-1 knockdown group by sigalectin-1 and the miR-22-3p-overexpressing group of the MDA-MB 436 cells. (**J**) Photographs of tumors harvested from mice injected with miR-22-3p-overexpressing clones, or galectin-1 knockdown clones of the MDA-MB 231 cells. (**K**) Volume measurements of tumors resulting from the miR-22 overexpression group (oemiR-22 group) or galectin-1 knockdown group (shgalectin-1). (**L**) Analysis of the mRNA levels of EMT-related proteins in the harvested tumor tissues using qRT-PCR. Significance was determined using an unpaired, two-tailed Student’s *t*-test when compared with the control. Data are presented as means ± SD. * *p* < 0.05, ** *p* < 0.01, and *** *p* < 0.001. EMT, epithelial-to-mesenchymal transition; qRT-PCR, real-time reverse transcription-polymerase chain reaction; SD, standard deviation.

Our cell experiments showed that the galectin-1 expression was regulated by miR-22-3p and that miR-22-3p overexpression and galectin-1 suppression inhibited the proliferation and invasion of breast cancer cells. To investigate the effects of miR-22-3p and galectin-1 on breast cancer progression, the proteins related to the EMT pathway and cell cycle were analyzed using Western blot. The MDA-MB-231 and T47D cells were transfected with miR-22-3p or sigalectin-1 and shgalectin-1. Interestingly, in the MDA-MB-231 cells, a triple-negative breast cancer cell line, the expression of galectin-1- and EMT-related markers Vimentin and Slug were significantly reduced by miR-22-3p overexpression (*p* < 0.05 and *p* < 0.05, respectively; Figure 7A,B). Compared with the MDA-MB-231 cells, the EMT markers induced by miR-22-3p overexpression in the T47D cells were not apparent, except for SMA (*p* < 0.05; Figure 7A,B). miR-22-3p is expected to control several targets related to EMT and galectin-1, and the EMT marker change after sigalectin-1 transfection was measured to confirm that the EMT marker change was mediated by galectin-1. The expression levels of Vimentin (*p* < 0.05; Figure 7D,E), Snail (*p* < 0.05; Figure 7D,E), and Slug (*p* > 0.05; Figure 7D,E) were reduced in the galectin-1 knockdown group of the MDA-MB-231 cells by sigalectin-1. Only Snail expression was decreased by sigalectin-1 in the T47D cells (*p* < 0.05; Figure 7D,E). The E-cadherin and SMA levels were not significantly affected by galectin-1 suppression. Slug expression was increased in the galectin-1 suppression groups of the T47D cells (Figure 7D,E). Significant changes in EMT markers induced by sigalectin-1 and miR-22-3p overexpression were more evident in the MDA-MB-231 cells compared with that in the hormone-positive T47D cells. We also confirmed the expression of EMT markers in the galectin-1 knockdown group of the MDA-MB-231 cells using shgalectin-1. The expression of Vimentin, Slug, and SMA was decreased in the galectin-1 knockdown group of MDA-MB-231 cells by shgalectin-1 (*p* < 0.05; Figure 7F,G). The expression of Snail was not altered by shgalectin-1, but was reduced by sigalectin-1 (Figure 7F,G). We confirmed that Vimentin, Slug, Snail, and SMA expressions were reduced in the galectin-1 knockdown groups of the MDA-MB-231 cells. We investigated whether the expression of Vimentin, Slug, Snail, and SMA was increased by galectin-1 overexpression, as opposed to the results of galectin-1 knockdown (Figure 7F,G). The expression of Slug, Snail, and SMA was seen as increasing patterns in the results of using galectin-1-overexpressing cells as opposed to the galectin-1 knockdown experiments (*p* < 0.05, *p* < 0.05, and *p* < 0.05; Figure 7F,G). The Vimentin level in the galectin-1 knockdown groups was decreased by siRNA and shRNA and increased by galectin-1 overexpression; however, the difference was not significant (Figure 7F,G). EMT-related proteins were also reduced in the experiments using the triple-negative MDA-MB 436 cells (Figure 7H,I). Galectin-1 expression was significantly decreased by miR-22-3p overexpression in the MDA-MB 436 cells (*p* < 0.05; Figure 7H,I). The expression levels of Snail, Slug, and SMA were decreased by miR-22-3p overexpression, and Slug expression was significantly reduced (*p* < 0.05; Figure 7H,I). However, the expression of Vimentin did not show much difference. Vimentin, Snail, Slug, and SMA were reduced in the galectin-1 knockdown group by sigalectin-1, of which Slug and SMA were significantly decreased (*p* < 0.01 and *p* < 0.05, respectively; Figure 7H,I). It is estimated that the inhibitory effect of miR-22-3p overexpression and galectin-1 suppression on EMT in the MDA-MB-231 and MDA-MB 436 cells is more pronounced than that in the T47D cells.

### 3.8. miR-22-3p Overexpression and Galectin-1 Knockdown Inhibit Tumor Growth and EMT Pathway In Vivo

Mouse xenograft models were established to explore the in vivo effects of miR-22-3p and galectin-1. We confirmed that the cell-proliferation rate, colony-formation rate, and invasion ability of the miR-22-3p overexpression clones and shgalectin-1 cells were reduced (Supplementary Figure S2, Figure 2). The miR-22-3p overexpression cell or stable knockdown cells of galectin-1 for MDA-MB 231 were implanted into the flank of nude mice via the subcutaneous injection of 5 × 10^6^ cells. The mice were sacrificed on days 25 and 33, and the tumors of different groups were harvested. Tumor growth in the miR-22-3p overexpression group (oemiR-22-3p group) was significantly lower than that in the control group (*p* < 0.05; Figure 7J,K). As with the miR-22-3p results, tumor growth in the galectin-1 knockdown group was also significantly decreased compared with that in the control group (*p* < 0.05; Figure 7J,K). Furthermore, the mRNA expression levels of galectin-1 were reduced in tumors from the miR-22-3p overexpression group and galectin-1 knockdown group compared with those in the control group (*p* < 0.001; Figure 7L). In addition, the mRNA expression levels of the EMT-related genes, Vimentin, Snail, and Slug, were significantly reduced in the miR-22-3p overexpression group (*p* < 0.001, *p* < 0.01, and *p* < 0.001, respectively; Figure 7L) and the galectin-1 knockdown group (*p* < 0.001, *p* < 0.001, and *p* < 0.001, respectively; Figure 7L) compared with those in the control group. These data suggest that miR-22-3p overexpression and galectin-1 knockdown inhibit tumor growth in vivo and are associated with the EMT pathway.

### 3.9. miR-22-3p Overexpression and Galectin-1 Knockdown Control Cell Cycle

**Figure 8:**
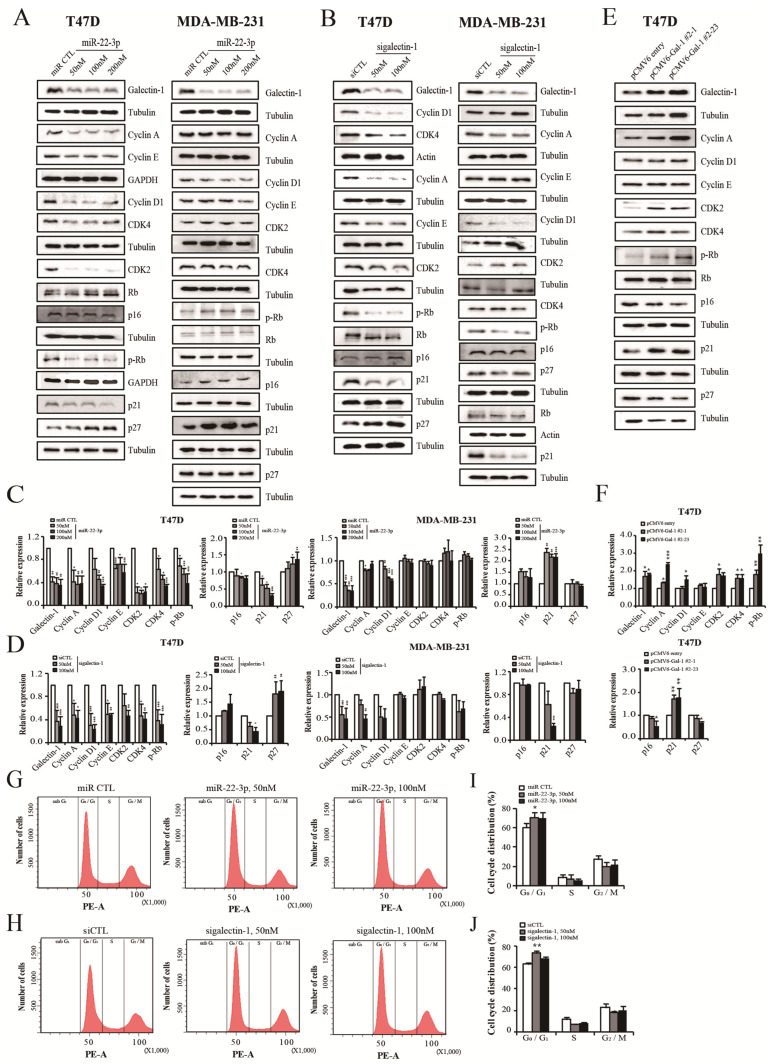
miR-22-3p overexpression and galectin-1 knockdown regulate the cell cycle. The expression of cell cycle regulatory proteins; cyclins A, D1, and E; and CDK2, CDK4, p16, p21, p27, Rb, and p-Rb was measured by Western blot after transfection with miR-22-3p and sigalectin-1. (**A**,**C**) Differential expression of cyclins A, D, and E, as well as CDK 2 and CDK4 in the miR-22-3p overexpression groups of the MDA-MB-231 and T47D cells. (**B**,**D**) Expression levels of cell cycle regulatory proteins in the galectin-1 knockdown groups of the MDA-MB-231 and T47D cells. (**E**,**F**) Expression of cell-cycle regulatory proteins in the galectin-1 overexpression cells of T47D. (**G**,**H**) FACS analysis after the transfection of miR-22-3p and sigalectin-1 of the T47D cells. (**I**,**J**) Analysis of the cell cycle-population changes induced by miR-22-3p and sigalectin-1 in the T47D cells. Significance was determined using unpaired, two-tailed Student’s *t*-test when compared with the control. Data are presented as means ± SD. * *p* < 0.05, ** *p* < 0.01, and *** *p* < 0.001. SD, standard deviation.

We also investigated the effects of miR-22-3p and galectin-1 on cell cycle progression and the analyzed cell cycle control proteins. Changes in the cell cycle-controlling factors were more significant in the T47D cells than in the MDA-MD 231 cells. The expression levels of cell cycle control factors were measured using Western blot after transfection with miR-22-3p and sigalectin-1 (Figure 8). The expression of cyclins (cyclin A, D1, and E) and cyclin-dependent kinases (CDK 2, CDK4) was significantly decreased in the miR-22-3p-overexpressing group of the T47D cells (*p* < 0.05; Figure 8A,C). In the MDA-MD 231 cell group, cyclin A and D1 levels were significantly decreased in the miR-22-3p overexpression group (*p* < 0.05 and *p* < 0.01, respectively; Figure 8A,C). CDK2 and CDK4 expression was not significantly altered in the miR-22-3p-overexpressing group of the MDA-MD 231 cells (Figure 8A,C). The expression levels of p16, p21, and p27, which are CDK inhibitors that regulate cyclin and CDK expression, were also confirmed. The expression of p21 and p27 revealed significantly decreasing (*p* < 0.05; Figure 8A,C) and increasing patterns (*p* < 0.05; Figure 8A,C), respectively, in the miR-22-3p overexpression group of the T47D cells. P16 expression was slightly decreased in the miR-22-3p overexpression group of the T47D cells. In the MDA-MB-231 cell groups, there were no significant changes in p27 expression. P21 expression was significantly increased in the miR-22-3p overexpression group (*p* < 0.01; Figure 8A,C). The expression of p16 slightly increased in the miR-22-3p overexpression group. The retinoblastoma protein (Rb) is a regulator of the cell cycle, and hyperphosphorylated Rb (p-Rb) induces the release of active E2F1 to drive G1 to S phase progression. A marked reduction in p-Rb expression was observed in the miR-22-3p overexpression group of the T47D cells (*p* < 0.05; Figure 8A,C). In the MDA-MB-231 cells, p-Rb expression slightly increased in the miR-22-3p overexpression group (Figure 8A,C). Significant reductions in p-Rb affected by cyclin reduction, CDK reduction, and increased CDK inhibitor in the miR-22-3p overexpression group of the T47D cells were expected to affect cell cycle progression. As a result, miR-22-3p was estimated to regulate several cell cycle control targets, which were measured after sigalectin-1 transfection to confirm whether the cell cycle was controlled by galectin-1. The expression of cell cycle controllers by galectin-1 knockdown in the T47D cells was consistent with the changes in the miR-22-3p overexpression group, which reduced cyclin A, D1, E, CDK2, CDK4, and pRb and increased p27 (*p* < 0.05; Figure 8B,D). Changes in cell cycle-related proteins caused by galectin-1 knockdown in the MDA-MB231 cells were relatively minimal, except for a slight decrease in cyclins A and D (*p* < 0.05; Figure 8B,D) indicating that cell cycle regulation was prominent in the T47D cells, a hormone receptor-positive breast cancer cell line. We established that the changes in cell cycle control factors induced by galectin-1 knockdown were reversed by galectin-1 overexpression in the T47D cells. The expression levels of cyclin A, D1, CDK 2, CDK4, and p-Rb were increased in the galectin-1-overexpressing cells of the T47D cells compared to the galectin-1 knockdown group (*p* < 0.05; Figure 8E,F). The expression of p27 was decreased and that of p21 was increased in galectin-1 overexpression cells (*p* < 0.01 and *p* < 0.05, respectively; Figure 8E,F). Changes in the cell cycle control factors were mainly observed in the T47D cells; therefore, a FACS analysis was conducted using the T47D cells. FACS confirmed that cell cycle progression was inhibited by miR-22-3p overexpression and galectin-1 knockdown in the T47D cells. A cell cycle analysis showed significantly increased numbers of G0/G1 phase cells (*p* < 0.05; Figure 8G,I) and reduced S and G2/M phase cell populations in the miR-22-3p overexpression group. The G0/G1 phase cell population also significantly increased (*p* < 0.01; Figure 8H,J), and the S and G2/M phase cell populations were reduced in the galectin-1 knockdown group of the T47D cells. These results indicated that miR-22-3p overexpression and galectin-1 knockdown may inhibit cell proliferation by arresting cell cycle progression in the G0/G1 phase. Therefore, these results suggest that miR-22-3p regulates the expression of several targets that can control the cell cycle and that galectin-1 regulates the cell cycle in hormone receptor-positive breast cancer and inhibits cancer progression.

## 4. Discussion

Galectin-1 has been studied as a therapeutic target in various types of cancer, including prostate, thyroid, colon, melanoma, bladder, ovarian, and breast cancers [18,19,20,21,25,26]. It plays a role in cancer progression through its involvement in cell migration and invasion, aggregation, adhesion to the ECM, angiogenesis, and apoptosis [18,19,20,21,22,23,24,25,26]. Herein, we report that galectin-1 knockdown can inhibit cancer progression in both hormone-positive and triple-negative breast cancer cells. And, its expression can be regulated by miR-22-3p, a small RNA. Galetin-1 knockdown inhibited cancer progression by regulating the cell cycle of hormone receptor-positive breast cancer cells and the EMT mechanism in triple-negative breast cancer cells. In addition, it was confirmed that miR-22-3p inhibits breast cancer growth by inhibiting galectin-1; however, it is estimated that miR-22-3p itself serves as a tumor suppressor through several targets. Galectin-1 is upregulated in breast carcinoma tissues and is clinically significant in patients with breast cancer [14]. Through the survival analysis of galectin-1 expression, its value as a prognostic factor has been established in prostate, ovarian, and stomach cancers, and high galectin-1 expression has been correlated with poor prognosis [21,40,41]. A survival rate analysis using the KM plotter showed that the survival rate was significantly lower in the high galectin-1 expression groups of hormone receptor-positive luminal type-B breast cancer and basal-type breast cancer.

In our study cohort, galectin-1 expression in breast cancer tissues was higher than that in control tissues and the MFS rate was lower in the high galectin-1 expression group; however, no significant difference was observed. Relatively few breast cancer tissues were analyzed in our study—more than 70% of the patients had hormone receptor-positive type breast cancer, and only nine were triple negative—which resulted in different findings from those obtained using the KM plotter.

RNA molecules are now at the center of molecular oncology, and their applications in diagnosis and therapy are being proposed. The ability of miRNAs to modulate important cellular processes by concurrently regulating multiple targets suggests their potential as therapeutic agents. We identified miR-22-3p, the most common miRNA, by searching for miRNAs likely to modulate galectin-1 expression using TargetScan, PicTar, miRanda, micro-T, and miRecords prediction programs, and demonstrated that miR-22-3p directly interacts with galectin-1. miRNAs are ncRNAs composed of 21–23 nucleotides that regulate gene expression after transcription. miR-22-3p expression, detected in several cancers, increases or decreases depending on the cancer type, and plays an essential role in various physiological processes and cancers via multiple targets. The expression of miR-22-3p varies depending on the type of cancer—it increases in prostate cancer and decreases in bladder, colon, hepatocellular, gastric, and breast cancers [31,32,33,34,42,43]. Several studies have identified numerous proteins targeted by miR-22 in breast cancer, including c-Myc-binding protein, Myc-associated factor X, and PTEN [35,36,37]. In addition, miR-22 inhibits estrogen signaling by directly targeting estrogen receptor alpha [38], and its overexpression suppresses growth and induces senescence-like phenotypes in human breast epithelial and cancer cells [39]. It has been reported that miR-22 acts as a tumor suppressor in breast cancer through several targets.

It has been proposed that miR-22-3p acts as an independent predictor of breast cancer outcome. Lymph node metastasis is a very powerful parameter of breast cancer prognosis. We found that miR-22-3p is significantly associated with lymph node metastasis in breast cancer, and patients with a low miR-22-3p level had a poorer survival rate than those with a higher level of miR-22-3p. Our results are consistent with those of Chen et al., who showed that miR-22-3p expression correlates with local relapse, distant metastasis, and survival in breast cancer [34]. In addition, our results showed the suppression of tumor growth and invasion by miR-22-3p, which supports its role as a tumor suppressor in breast cancer cells.

Few studies have investigated the role of galectin-1 as a target for miR-22-3p in cancer. The association between galectin-1 and miR-22-3p has been reported in hepatocellular carcinoma (HCC) [44]. Studies on HCC have shown that galectin-1 is negatively regulated by miR-22-3p, indicating its potential as a prognostic marker and therapeutic target in HCC. These results are consistent with our findings in breast cancer cells. Galectin-1 is one of several targets of miR-22-3p, and galectin-1 expression is regulated by miR-22-3p in breast cancer cells. Cell proliferation, migration, and invasion were inhibited by galactin-1 knockdown and miR-22-3p overexpression in both hormone receptor-positive and basal-like breast cancer cells. These results suggest that galectin-1 and miR-22-3p are involved in breast cancer progression through interactions and that the regulation of the galectin-1/miR-22-3p network could be a treatment strategy.

The RNAi phenomenon is a method of inhibiting the expression of genes by silencing their target genes. There are two main types of RNA-inducing substances, siRNAs and miRNAs, which enable the selective degradation of specific target mRNAs. Using gene silencing, new treatments are being developed to overcome not only cancer but also various genetic diseases. Nishimura et al. demonstrated sandwich RNAi inhibition by targeting the important ovarian cancer oncogene, EphA2, using a combination of EphA2-targeting siRNAs and miR-520d-3p (an EphA2-targeting miRNA) mimics. This dual targeting of EphA2 showed elevated antitumor efficiency compared to that induced by two single therapies [45].

In this study, the use of siRNA and miRNA combination treatments (sigalectin-1/miR-22-3p) showed better antitumor effects than the single treatments. Thus, regimens using a cocktail of RNAi-based therapeutics to target dominant oncogenes may achieve improved therapeutic outcomes in human cancers.

Among the various roles of galectin-1 in cancer, cell proliferation has been reported to act differently depending on cancer cell type, tumor immune privilege, and galectin-1 interactions with specific cell-surface glycoproteins or intracellular proteins [46]. Galectin-1 suppression inhibits cell growth in mouse mammary tumor cells and in head and neck, lung, cervical, and ovarian cancers, and exogenous galectin-1 induces the proliferation of ovarian and pancreatic cancer cells [47,48,49,50,51,52,53]. Other studies have shown that NCAPG interacts with LGALS1 to promote the proliferation, invasion, and migration of NSCLC cells [54].

Conversely, galectin-1 reduces cell proliferation in colon cancer and HCC by inducing p27 and p21 [55]. The same result for galectin-1—inhibiting cell growth—was observed in human neuroblastoma cells, wherein galectin-1 interacted with ganglioside GM1, thereby disturbing cell–cell and cell–stroma interactions and inhibiting cellular proliferation [56]. It has also been reported that galectin-1 regulates cell growth and metastasis in breast cancer. In a study using mouse mammary cancer cells, the silencing of galectin-1 expression decreased immunosuppressive activity and suppressed tumor growth and lung metastasis [57]. Conversely, other studies have shown that galectin-1 inhibits cell proliferation and metabolic activity, and induces apoptosis in MCF-7 breast cancer cells [58].

In our study, miR-22-3p overexpression and galectin-1 knockdown suppressed the growth and invasion of breast cancer cells. Cell cycle control factors and EMT-related molecules, which are important in cancer progression, were also expressed in a direction that suppressed tumor growth and invasion. In this study, the expression of the tumor-suppressive CDK inhibitor p27 was increased, and the expression of oncogenic CDK2 and CDK4; cyclins (A, D, and E); and pRb was decreased by galectin-1 knockdown and miR-22-3p overexpression in primary hormone receptor-positive breast cancer cells. The expression of cyclins A and D was decreased, but changes in cell cycle-related factors were not evident in triple-negative cancer cells compared with those in hormone receptor-positive breast cancer cells. The FACS analysis confirmed that cell cycle progression was inhibited by the arrest of the cell cycle in the G0/G1 phase by galectin-1 knockdown and miR-22-3p overexpression in the T47D cells.

Therefore, it is estimated that the regulation of galectin-1/miR-22-3p expression inhibits tumor growth by regulating the cell cycle in hormone receptor-positive breast cancer. Currently, CDK inhibitors Palbociclib, Avemaciclib, and Ribociclib are used as clinical therapeutic drugs in hormone receptor-positive breast cancer therapy. Thus, the regulation of galectin-1/miR-22-3p expression is expected to play an important role in the treatment of hormone receptor-positive breast cancer, and many therapeutic targets may be selected from among the cell cycle regulatory molecules identified in our results.

EMT is a process in which epithelial cells lose their epithelial cell properties and cell-to-cell contacts, increasing the invasiveness of cancer, and plays an important role in tumor progression and metastasis. Galectin-1 is related to the EMT pathway in several carcinomas. Galectin-1 is involved in tumor invasion, metastasis, and EMT as it controls the expression of MMP-2, MMP-9, and TIMP [59,60]. Studies on ovarian cancer have shown that the downregulation of Galectin-1 with siRNA-based strategies increases the mRNA levels of E-cadherin and decreases the levels of N-cadherin, MMP7, fibronectin, Snail, and Slug, thereby inhibiting the EMT pathway [61].

In our study, the expression levels of Vimentin, Snail, and Slug, which act primarily on the EMT pathway, were decreased by the miR-22-3p overexpression and galectin-1 suppression by siRNA in triple-negative breast cancer cells. Changes in EMT-related molecules were not significant in the hormone receptor-positive cells. The expression of Vimentin, Snail, and Slug was significantly reduced in the tumor tissues obtained through xenografts using miR-22-3p stable cells and galectin-1 knockdown cells. Therefore, the regulation of the galectin-1/miR-22-3p axis is expected to be an important therapeutic target via EMT regulation in triple-negative breast cancer treatment.

This study had several limitations, including the small number of breast tissues that were used for clinical analysis; more than 70% of these were hormone receptor-positive and only nine were triple-negative type, which made the analysis depending on the molecular type difficult and different from the KM plotter results. Changes in cell cycle control factors were mainly observed in the T47D cells. However, miR-22-3p also regulated the expression of cell cycle control targets in the MDA-MB 231 cells. Even when galectin-1 was inhibited, cyclin D1/A was reduced; therefore, it can be assumed that cell cycle control is minimal in MDA-MB 231 cells. In addition, EMT relevance was mainly observed in the MDA-MB 231 cells; however, it can be estimated that in T47D cells, Snail expression is reduced when galectin-1 is inhibited, resulting in minimal EMT relevance. However, further clarification of the underlying mechanism is required.

The study revealed that the miR-22-3p/galectin-1 expression axis is associated with prognosis in breast cancer patients, with high miR-22-3p expression and low galectin-1 expression being associated with favorable prognosis. We also identified that miR-22-3p, with prognostic significance, directly interacts with the 3′ UTR of galectin-1, leading to a suppression of its expression. Furthermore, the downregulation of galectin-1 functions as a tumor suppressor, influencing the cell cycle and EMT pathways in breast cancer to mitigate tumor progression. The most remarkable finding from this study is that the miR-22-3p/galectin-1 axis affects cancer progression by regulating the cell cycle in hormone receptor-positive breast cancers and by affecting EMT mechanisms in triple-negative breast cancers. The molecular mechanisms by which the miR-22-3p/galectin-1 axis is involved in cancer progression need to be further investigated.

In conclusion, our findings demonstrate that targeting the miR-22-3p/galectin-1 axis, which acts as a tumor suppressor and is associated with prognosis in breast cancer, may be a promising therapeutic strategy and may offer the possibility of tailoring treatment according to the molecular subtype of breast cancer.

## Figures and Tables

**Table 1:** Differential expression of miR-22-3p according to tumor characteristics.

Tumor Characteristics		Number	Mean ± SD	*p* Value
		54	0.0427 ± 0.0337	
T stage	1	25	0.0409 ± 0.0314	0.554
	2–3	29	0.0464 ± 0.0366	
N stage	0	31	0.0553 ± 0.0396	0.001
	1–3	23	0.0284 ± 0.0147	
TNM stage	1	16	0.0471 ± 0.0357	0.110
	2	21	0.0526 ± 0.0409	
	3	17	0.0299 ± 0.0341	
Estrogen receptor	Negative	22	0.0370 ± 0.0209	0.178
	Positive	32	0.0485 ± 0.0404	
Progesterone receptor	Negative	17	0.0405 ± 0.0208	0.545
	Positive	37	0.0454 ± 0.0388	
HER-2/neu status	Negative	44	0.0451 ± 0.0371	0.376
	Positive	10	0.0385 ± 0.0149	

**Table 2:** Tumor characteristics and metastasis-free survival.

Tumor Characteristics		Mean MFS (m, 95% CI)	Log Rank
T stage	1	114.50 (103.47–125.53)	0.428
	2–4	108.74 (91.79–125.70)	
N stage	0	124.14 (117.71–130.58)	0.005
	1–3	96.0 (75.85–116.19)	
Estrogen receptor	Negative	106.27 (86.67–125.87)	0.328
	Positive	114.98 (103.68–126.30)	
Progesterone receptor	Negative	100.57 (78.10–123.04)	0.070
	Positive	116.21 (105.65–126.78)	
HER-2/neu status	Negative	119.10 (108.26–129.95)	0.030
	Positive	84.60 (54.66–114.55)	
miRNA-22-3p level	Low	105.37 (89.98–120.78)	0.040
	High	123.33 (116.39–130.28)	
Galectin-1 level	Low	118.09 (104.45–131.74)	0.253
	High	92.60 (79.30–105.90)	

## Data Availability

The data that support the findings of this study are available upon request from the corresponding author.

## Supplementary Materials

The following supporting information can be downloaded at https://www.mdpi.com/article/10.3390/cells14040310/s1, Figure S1. Dose–response curve of miR-22-3p, sigalectin-1, and combined miR-22-3p and sigalectin-1 in the MDA-MB 231 and T47D cells. Cells were treated with different concentrations of miR-22-3p, sigalectin-1, or a combination of miR-22-3p and sigalectin-1 in a 1:1 ratio for 72 h followed by MTT analysis (A) and analysis of invasion ability (B). Figure S2. Functional analysis of miR-22-3p overexpression stable clones. (A) The expression level of miR-22-3p was determined using quantitative RT-PCR and the expression of galectin-1 was determined using Western blot. (B) Proliferation assay, (C) colony forming assay, (D) invasion assay, and (E) wound healing assay of miR-22-3p overexpression clones. qRT-PCR, real-time reverse transcription-polymerase chain reaction. Table S1. Primary antibody used.
