# Supplementary material for: Therapeutic Targeting of the Galectin-1/miR-22-3p Axis Regulates Cell Cycle and EMT Depending on the Molecular Subtype of Breast Cancer
Source: Cells. 2025 Feb 19;14(4):310. doi: 10.3390/cells14040310 (PMC11854374; doi:10.3390/cells14040310)
Supplement: Supplementary file 1 [file cells-14-00310-s001.zip › cells-3437591-supplementary.pdf]

Table S1. Primary Antibody used

Antibody	Species	Dilution	Company (Catalog#)
Galectin-1	Rabbit	1:1000	Sigma, HPA000646
Vimentin	Mouse	1:1000	Santa cruz biotechnology, sc-73259
Slug	Mouse	1:1000	Abcam, ab51772
Snail	Rabbit	1:500	Abcam, ab216347
SMA	Mouse	1:1000	Abcam, ab7817
E-cadherin	Mouse	1:1000	Santa cruz biotechnology, sc-21791
CyclinA	Mouse	1:500	Santa cruz biotechnology, sc-274682
CyclinD1	Mouse	1:500	Santa cruz biotechnology, sc-20044
CyclinE	Mouse	1:1000	Santa cruz biotechnology, sc-377100
CDK2	Mouse	1:500	Santa cruz biotechnology, sc-6248
CDK4	Mouse	1:500	Santa cruz biotechnology, sc-56277
p-Rb	Rabbit	1:1000	Cell signaling, #9301
Rb (total)	Mouse	1:500	Santa cruz biotechnology, sc-102
p16	Rabbit	1:1000	Abcam, ab51243
p21	Rabbit	1:1000	Cell signaling, #2947
p27	Rabbit	1:1000	Cell signaling, #3686
α-Tubulin	Mouse	1:1000	Santa cruz biotechnology, sc-5286
β-Actin	Mouse	1:1000	Santa cruz biotechnology, sc-47778

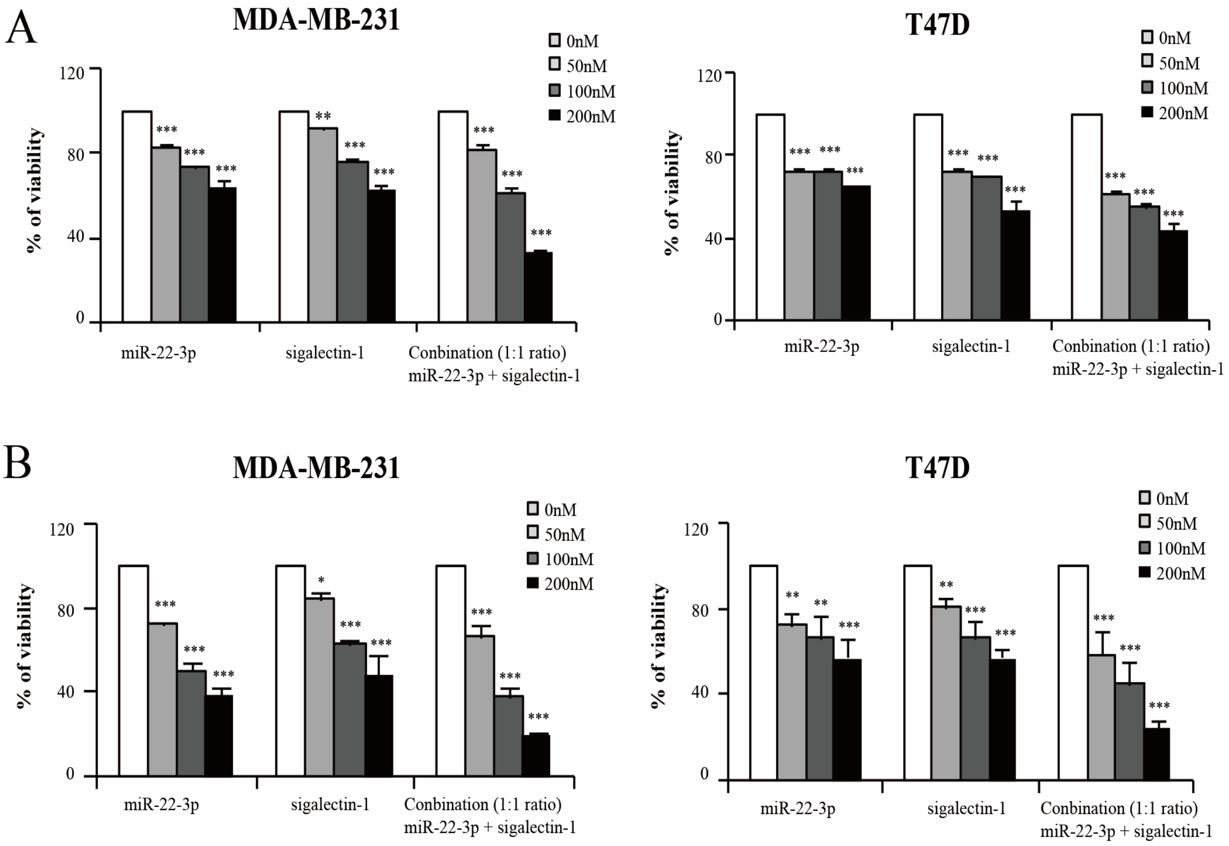

Figure S1. Dose-response curve of miR-22-3p, sigalectin-1, and combined miR-22-3p and sigalectin-1 in MDA-MB 231 and T47D cells. Cells were treated with different concentrations of miR-22-3p, sigalectin-1, or a combination of miR-22-3p and sigalectin-1 in a 1:1 ratio for 72 h followed by MTT analysis (A) and analysis of invasion ability (B).

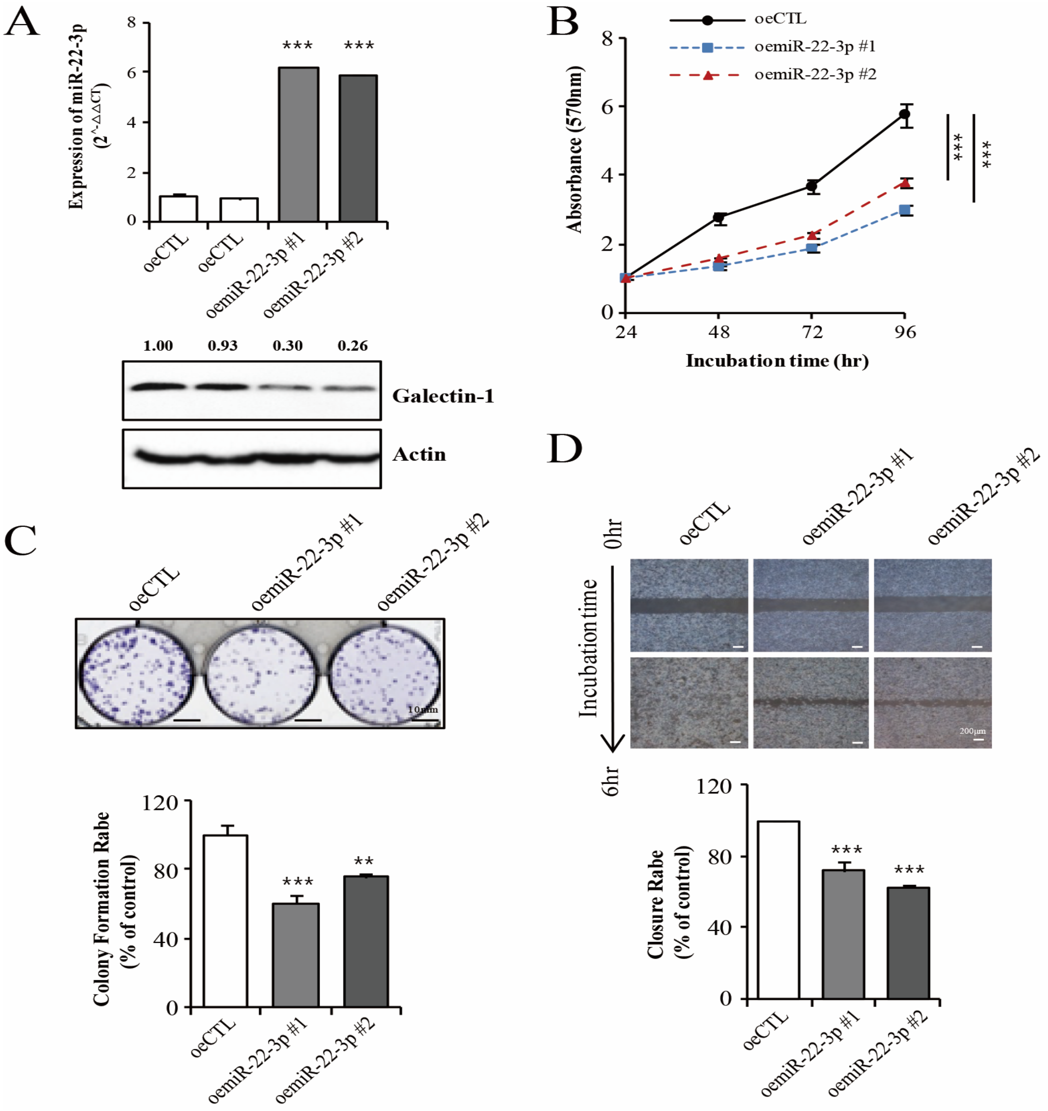

Figure S2. Functional analysis of miR-22-3p overexpression stable clones. (A) The expression level of miR-22-3p determined using quantitative RT-PCR and expression of galectin-1 determined using western blot. (B) Proliferation assay, (C) colony forming assay, (D) invasion assay, and (E) wound healing assay of miR-22-3p overexpression clones. qRT-PCR, real-time reverse transcription-polymerase chain reaction
